# Integrating machine learning for advanced analysis of bioelectrical impedance parameters in children with nephrotic syndrome

**DOI:** 10.3389/fped.2026.1714324

**Published:** 2026-02-16

**Authors:** Josephine Reinert Quist, Leigh C. Ward, Lars Jødal, René Frydensbjerg Andersen, Christian Lodberg Hvas, Steven Brantlov

**Affiliations:** 1 Department of Hepatology and Gastroenterology, Aarhus University Hospital, Aarhus, Denmark; 2 School of Chemistry and Molecular Biosciences, The University of Queensland, Brisbane, QLD, Australia; 3 Department of Nuclear Medicine, Aalborg University Hospital, Aarhus, Denmark; 4 Department of Paediatrics and Adolescent Medicine, Aarhus University Hospital, Aarhus, Denmark; 5 Department of Clinical Medicine, Aarhus University, Aarhus, Denmark; 6 Department of Procurement & Clinical Engineering, Aarhus University Hospital, Aarhus, Denmark

**Keywords:** bioelectrical impedance, children, electric capacitance, machine learning, oedema

## Abstract

**Background:**

Nephrotic syndrome (NS) in children, characterised kidney-related protein leakage and peripheral oedema, remains challenging to assess. Bioelectrical impedance analysis (BIA) provides indices of body water (oedema), and analysis with machine learning (ML) may improve clinical care. We tested an ML model to identify NS in children compared with healthy children.

**Methods:**

This cross-sectional study included children with active NS in the acute phase (aNS group) recruited from the Department of Paediatrics and Adolescent Medicine, Aarhus University Hospital, Denmark. Anonymised MF-BIA data from frequencies between 5 and 1000 kHz were analysed using the web-based ML platform JustAddDataBio (JADBio)® to identify potential biomarkers for improved diagnosis.

**Results:**

Eight children with aNS and 38 healthy children of similar ages were included. The ML software employed ridge logistic regression with the penalty hyperparameter lambda = 0.001 and a selected threshold of 0.81 by JADBio. The best model achieved an area under the curve (AUC) of 0.84 [95% confidence interval (CI): 0.72;0.94]. The software selected the following features: height, age, resistance at 50 kHz, impedance at 50 kHz, the characteristic frequency, phase angle at 50 kHz, and sex. The model demonstrated a statistically significant true positive classification rate of 0.92 (92%) [CI: 0.88;0.96] and a specificity of 0.22 (22%) [CI: 0.08;0.36].

**Conclusion:**

Applying ML-supported evaluation of BIA affirmed diagnostics. However, low specificity limits clinical applications. A larger population of patients and inclusion of additional biomarkers may be needed to develop a more acceptable model.

## Introduction

Nephrotic syndrome (NS) is a relatively rare disease, with an incidence of 2–7 per 100,000 children (1). It is characterised by excessive renal protein loss, leading to hypoalbuminemia and oedema (2). It is often associated with extended hospitalisation (3). Conventionally, the degree of oedema is estimated through clinical evaluation (4) and body weight measurement, where weight gain is assumed to primarily indicate accumulation of extracellular water (ECW). However, this approach has limitations, particularly over extended periods when weight changes due to factors other than ECW become significant (5).

In the search for a practical, cost-effective, and non-invasive technique to evaluate routine oedema in children with NS, bioelectrical impedance analysis (BIA) has emerged as a promising tool (6, 7). BIA offers characteristics ideally suited for clinical routine use, such as safety, speed, simplicity, and portability (8). BIA operates on the principle that the flow of alternating current through the body varies across tissues. Tissues rich in water and electrolytes exhibit high conductivity and low impedance (Z, ohm), while fat and bone, with low conductivity, show high impedance. Impedance comprises two components: resistance (R) and capacitive reactance (X_C_). The body's resistance is inversely related to its water content (R∼1/body water), which is the foundation for estimating body water volumes with BIA (9). BIA devices vary in measurement capability: Some use only a single frequency (SF-BIA), while others use multiple frequencies (MF-BIA). The bioimpedance spectroscopy (BIS) technique measures a broad spectrum of frequencies and employs the Cole model for data analysis, where coordinate points (R, X_C_) are plotted and fitted to a semicircular arc to describe the data.

From BIA measurements, clinical body parameters such as ECW, total body water (TBW), fat-free mass, and fat mass can be estimated using prediction models derived from healthy individuals. Importantly, these estimates rely on assumptions that may vary across study populations. Prediction equations typically vary among BIA devices from different manufacturers, and only some manufacturers disclose the specifics of their equations.

To overcome such transparency-related limitations, increasing attention has been given to the use of the primary measured electrical properties, which are openly described and independent of manufacturer-specific BIA devices. Such parameters are referred to as “raw” or “derived raw” impedance parameters, acting as proxies for their physiological correlates. We use the term “derived raw” to reflect that these parameters are not directly measured, but rather the results of basic, theoretically based calculations applied to the measured data.

Four important derived raw impedance parameters are the phase angle (PhA), impedance ratio (IR, ratio of impedances measured at different frequencies, typically a high and a low frequency), cell membrane capacitance (C_m_), and resistance (R). PhA, IR, and C_m_ are composite parameters derived from various raw data, whereas R directly reflects opposition to the flow of electric current through the body's tissues, influenced predominantly by the body's total water content and tissue composition. Each of these parameters may be valuable for assessing body water distribution in NS patients. PhA is associated with tissue hydration, cell membrane quality, and cellular mass (10–12), although its exact biological significance remains under ongoing investigation (13). IR is another promising parameter that potentially indicates oedema or overall health, reflecting fluid distribution between TBW and the ECW. Lastly, C_m_ provides insights into cell membrane function, size, hydration status, and overall tissue composition (14, 15). Although research is being conducted into the utility of these parameters—particularly PhA in adults—there is a scarcity of studies focusing on paediatric populations (16).

Integrating machine learning (ML) offers capabilities such as efficient processing of large datasets, precise pattern recognition, and predictive modelling (17). These features increase the ability of clinicians to extract intricate insights from impedance measurements, enhancing their understanding of parameters like PhA, IR, and C_m_ in NS. ML's notable speed and ability to minimise mean prediction error may ensure timely and informed decision-making, facilitating more effective patient care and treatment strategies.

The aim of this clinical pilot study was to assess the feasibility of employing ML software with bioimpedance outcomes—PhA, IR, C_m_, and R—to effectively identify and differentiate children with aNS, thereby contributing to further characterisation of body composition.

## Materials and methods

### Study design

This was a single-centre cross-sectional study, reported in accordance with the STREAM-URO network guidelines (18).

### Study subjects

Children aged 3–10 years with active NS in the acute phase (aNS patient group) were included from the Department of Paediatrics and Adolescent Medicine, Aarhus University Hospital, Denmark. Prior to initiation of treatment with prednisolone and diuretics, blood samples, blood pressures measurements, and impedance assessments were collected from the patient group. Patient data were obtained from a previously described cohort (2). The healthy control group consisted of 38 children selected from a previously established reference cohort of children, chosen to span a representative range of age and sex, but not individually matched to the patients with NS (19). Results are presented as mean ± standard deviation (SD), after testing for normality using Q–Q plots and the Shapiro–Wilk test.

### BIA device and parameters

Whole-body impedance was measured using electrodes placed on the wrist and ankle in pairs. A Xitron 4200, HYDRA BIS device (Xitron Technologies, San Diego, CA, USA) was employed and tested weekly with an electronic verification module (TS4201) according to the manufacturer's instructions. The BIS device performed measurements of electrical parameters, including impedance (Z), resistance (R), and capacitive reactance (X_C_), all measured in ohms (Ω), at 50 different frequencies ranging from 5 to 1,000 kHz.

Impedance (Z) is defined as the length of the vector to an individual point, $Z=\sqrt{R^{2}+X_{C}^{2}}$. PhA is the angle between the vector and the horizontal axis. For measurements at a given frequency, the phase angle is calculated as PhA = tan^−1^(X_C_/R) · 180 °/π. PhA depends on the frequency but is often reported at 50 kHz (3, 19). This requires only SF-BIA (the single frequency being 50 kHz). When full-spectrum data are available (BIS), PhA may also be reported at the characteristic frequency *f*_c_, corresponding to the frequency of maximum reactance.

Impedance ratio, IR, compares impedance at a high frequency with the impedance at a low frequency. Measurements at high frequencies are dominated by TBW, while measurements at low frequencies are dominated by ECW; typically, the frequencies used for IR are 200 and 5 kHz (20). IR is calculated as IR_200/5_ = R_200_/R_5_ and has no unit (3). Determination of IR requires at least two measurements, i.e., MF-BIA or BIS.

Cellular membrane capacitance, C_m_, is calculated from the Cole model based on the spectrum of frequencies (20). The unit of C_m_ is nanofarad, nF. Determination of C_m_ requires knowledge of a spectrum of frequencies, i.e., BIS not SF-BIA or MF-BIA.

### Data acquisition

Prior to conducting impedance measurements, trained personnel recorded weight and height in duplicate. Weight, measured on digital scales with light clothing, was recorded to the nearest 0.1 kg, while height, measured without shoes using a stadiometer, was recorded to the nearest 0.5 cm.

BIA measurements adhered to standardised procedures (6, 7), with participants refraining from intense physical exercise for 4 h before the study but not required to fast. Measurements were performed in an electrically neutral environment. Participants rested for 5 min before measurement on a non-conductive surface. Arms and legs were positioned at a 35–40° angle from their trunk. The skin areas, where  > 4 cm^2^ ECG-style gel electrodes were to be applied, were cleaned with alcohol prior to attachment. The device cables were kept from touching the subject, parents, any nearby objects including metal and the technical equipment. Measurements were repeated three times with the electrodes remaining in the same place. The measurements were performed between 8.30 a.m. and 3.30 p.m. Details of the protocol can be found in a previous publication (6) and in the studies from which the subjects were initially enrolled (2, 19). Data were extracted using the ImpediMed SFB7 Multi-Frequency Analysis software (Bioimp Version 5.4.0.3, Brisbane, QLD, Australia) from measurements at 50 kHz and at the characteristic frequency (*f*_c_), where capacitive reactance is at its maximum value. These measurements were used to calculate specific parameters within the software.

In this study, the parameters analysed at 50 kHz were resistance (R_50_), capacitive reactance (X_C50_), impedance (Z_50_), and phase angle (PhA_50_). The parameters analysed at the subject-specific characteristic frequency were resistance (R*_f_*_c_), capacitive reactance (X_C*f*c_), impedance (Z*_f_*_c_), and phase angle (PhA*_f_*_c_). In addition, C_m_ was also analysed. Each subject was measured three times in one setting, and the mean values from the three measurements were used as individual data points for input to the software. Finally, clinical data such as sex, height, weight, and age were included.

### Machine learning software

**Figure 1:**
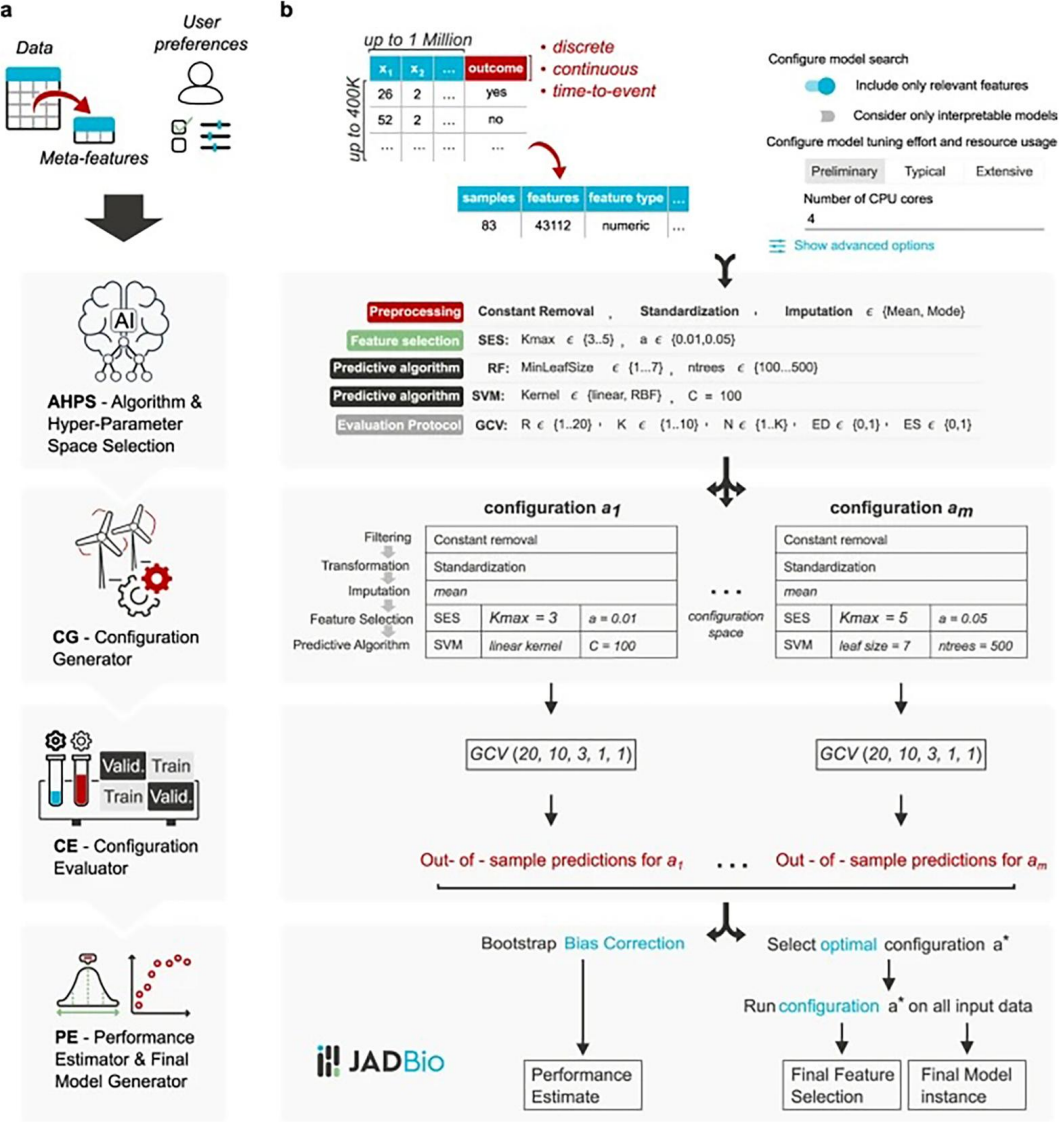
JADBio software illustration. Reproduced from Tsamardinos et al. (21) “Just add data: automated predictive modelling for knowledge discovery and feature selection,” licensed under CC BY 4.0. JADBio architecture. Panel **(a)** visualizes the architecture at a high-level, while panel **(b)** visualizes the details. The Algorithm and Hyper-Parameter Space selection system (AHPS), analyzes the dataset meta-features (characteristics of the data such as sample size) and user preferences. Based on them, it selects the appropriate combinations of algorithms and hyper-parameter values to try which form the configuration space to explore. It also decides the hyper-parameters of the generalized cross validation (GCV) algorithm, i.e., the protocol for estimating each configuration's performance. Based on these decisions, the Configuration Generator (CG) instantiates the configurations to be evaluated. The Configuration Evaluator (CE) identifies the winning configurations using generalized cross-validation (GVC), i.e., by repeatedly applying each configuration on different partitions of the data (“Train” boxes) to train a model and estimating performance on the remaining data (out-of-sample predictions, “Validation” boxes). The Performance Estimator and Final Model Generator (PE) produces the final model and its performance. It pools together all out-of-sample predictions during cross-validation and feeds them to the Bootstrap Bias Correction algorithm. This is to remove the bias due to trying multiple configurations. The final model is produced on all available data using the winning configuration.

Just Add Data Bio (JADBio)® (3154 Glendale Blvd Los Angeles, CA 90039-1830, USA) (21) is a web-based machine learning platform designed for analysing potential biomarkers to diagnose and estimate the prognosis of a disease. It is a fully automated machine learning software developed for small sample populations with many data points for each subject. It was designed for predictive modelling and exploring possible machine learning applications with minimal entry-level skills typically required for developing such models. Once data are uploaded into the JADBio software, it starts locating the relevant features (e.g., variables that help determine the investigated outcome) and returns the developed model and information related to it. It comprises several algorithms across the standard phases of machine learning model construction—data preprocessing, data transformation, data imputation, feature selection, and predictive modelling—as shown in Figure 1 (Reproduced from Tsamardinos et al. (21) “Just add data: automated predictive modelling for knowledge discovery and feature selection,” licensed under CC BY 4.0). The label of interest was disease activity, where “false” indicated a child without aNS and “true” indicated a child with aNS. Data were divided into training and testing cohorts.

In data pre-processing, missing values were imputed using mean and mode imputation, whereby the mean of the observed values was used to replace missing data. Zero-variance features were removed, as they provided no discriminatory information for predictive modelling. Continuous features were standardised to zero mean and standard deviation (SD) of 1. The imputation of missing data for a variable replaced the missing value with an estimate derived from the distribution of the variable (22). If imputation was not possible, the missing data and the reasons behind it were disclosed. Feature selection algorithms—least absolute shrinkage and selection operator (LASSO) regularised regression and statistical equivalent signatures—were investigated to determine the minimal size predictive feature subset. Predictive modelling algorithms were tested, including decision trees, ridge logistic regression, random forests, support vector machines, cox regression, and random survival forests (21). Algorithm choice was determined by the software algorithm and hyperparameter space selection system, which is an AI decision support system. The software does not provide a rational for the different algorithms used. Removal of features tested for a machine learning model was guided by clinical intuition and research group discussion, based on assumptions that increase the chance for causal inference (23). Performance estimations were based on generalised cross-validation (CV), which determined the hyperparameters. These hyperparameters are external settings for a model that influence its performance (e.g., learning rates and regularisation strengths). Cross-validation is a technique in which the sample size is proportionally divided into K folds of equal size. K refers to the number of groups into which a given data sample will be split. In R-repeated cross-validation, the procedure is run R times with different partitions (referring to the divisions or subsets into which the dataset is divided) into folds, thereby reducing variance in the estimation. The final model was estimated with a bootstrap bias correction to adjust *p*-values and to remove bias due to the multiple tries. Bootstrapping, a statistical technique involving resampling with replacement from observed data, estimates uncertainty in sample statistics. In the final model estimation, bootstrap bias correction was applied to adjust *p*-values, mitigate bias resulting from multiple attempts, and increase the risk for individual error (24). The working model was evaluated using sensitivity, specificity, accuracy, receiver operating characteristic (ROC) curves for correct classification of children with or without aNS, false positive rate, and true positive rate. This model can be changed with different thresholds for the ROC. Predictive performance was defined as the average accuracy across the test folds in a repeated ninefold cross-validation. Individual conditional expectation (ICE) plots visualised the effect of a given variable for a specific model. The final analysis was evaluated by the area under the curve (AUC) and CI for the best model.

## Results

### Patient characteristics

Data were collected from eight children with aNS—seven boys and one girl—and 38 healthy controls (HC group)—23 boys and 15 girls—obtained from a previous study reported by Brantlov et al. (2). Of the eight children with aNS, five experienced remission defined by no protein in a urine sample for 3 consecutive days. These five children out of the original eight were re-measured and subgrouped in a remission group (NSr). Patients with aNS had a mean age of 6.7 ± 3.1 years, the subgroup patients with NSr had a mean age of 7.7 ± 3.8 years, and the HC had a mean age of 7.5 ± 2.2 years. Children with aNS and healthy controls had comparable body weights, and all participant characteristics are summarised in Table 1. Bioimpedance data are presented in Table 2.

**Table 1:** Characteristics of the subjects enrolled in the study.

Parameter	ANS	ANS*	NSR	HC
Sex (M/F)	7/1	4/1	4/1	23/15
Age (years)	6.9 ± 3.1	6.8 ± 3.1	7.7 ± 3.8	7.5 ± 2.2
Study weight (kg)	31.3 ± 17.1	28.5 ± 9.6	28.3 ± 10.4	25.3 ± 6.2
Height (cm)	120.7 ± 21.1	120.3 ± 21.8	126.1 ± 26.2	126.4 ± 14.2
BMI (kg/m^2^)	20.1 ± 4.6	19.1 ± 1.5	17.3 ± 2.0	15.6 ± 1.2

Data are means ± SD. aNS, active nephrotic syndrome; aNS*, patients who entered remission; NSr, patients re-measured at remission; HC, healthy controls.

**Table 2:** Bioimpedance data of enrolled subjects.

Parameter	aNS	aNS*	NSr	HC
R_E_ (ohm)	447.1 ± 48.9	420.2 ± 43.5	752.6 ± 71.7	816.3 ± 73.8
R_I_ (ohm)	1,871.6 ± 182.3	1,919.7 ± 176.2	1,799.5 ± 239.5	1,922.3 ± 224.0
R_INF_ (ohm)	359.7 ± 33.3	344.1 ± 31.6	528.6 ± 46.0	572.3 ± 53.2
C_m_ (nF)	0.53 ± 0.21	0.47 ± 0.13	0.82 ± 0.34	0.68 ± 0.20

Measured resistances (R) and cell membrane capacitances (C_m_) in the study. Data are means ± SD. R_E_, resistance at 0 kHz (resistance of extracellular water); R_I_, resistance of intracellular water; R_INF_, resistance at infinite frequency (resistance of total body water); C_m_ (nF), Celle membrane capacitance; aNS, active nephrotic syndrome; aNS*, patients who entered remission; NSr, patients re-measured at remission; HC, healthy controls.

### Data abstraction

BIA data were obtained from the eight children with aNS. Only five who had achieved remission and were re-measured were included in the software analysis as “healthy,” together with the HC group. The three patients who were not in remission/unknown status and consequently not re-measured, and therefore not included in the NSr group, had either experienced repeated relapses (two children) or been transferred to another hospital (one child). The software had 2,507 data points, with 51 data points missing.

Initially, the JADBio first ML model identified body weight as the only influential feature, prompting its exclusion from the dataset based on a discussion in the group before execution of the second model. This was to decrease the bias provided by the weight feature, which would obscure other potential features for a model.

### Model specification

**Figure 2:**
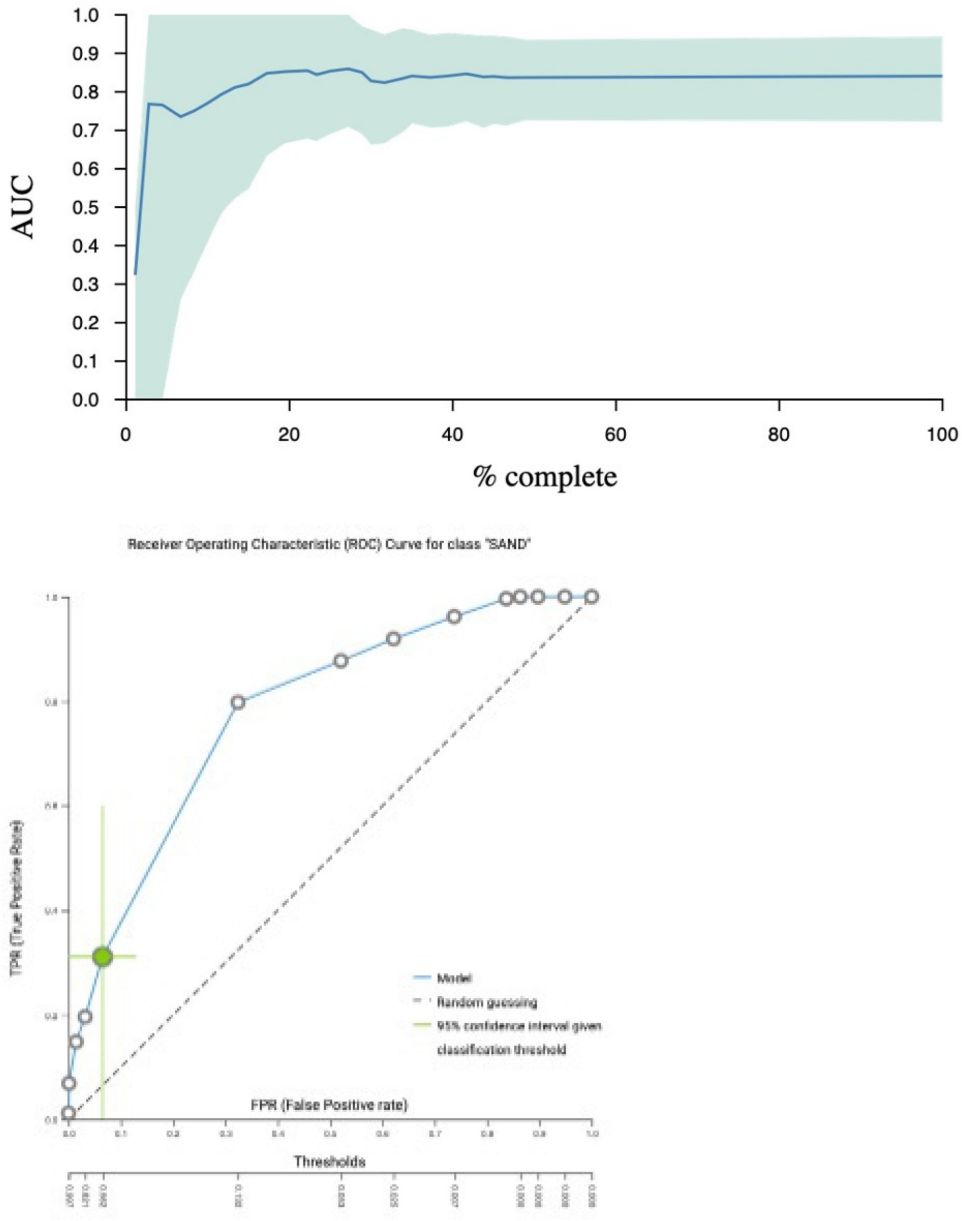
AUC curve illustrated with an AUC = 0.84, meaning that in 84% of the cases, it can distinguish between healthy controls and patients. ROC plot with the threshold of 0.82, meaning if the model has an output >0.80, it is a positive case.

For classifying the eight children with aNS compared to the 38 HC, bootstrapping of the real-world data generated a large number of models (271,530 models with 3,017 configurations) that were trained and explored using an extensive tuning effort. The best model for overall performance (e.g., precision and true positive rate) with the chosen threshold was ridge logistic regression with the penalty hyperparameter lambda = 0.001, with an AUC of 0.84 (CI: 0.72; 0.94) and a threshold of 0.81. See Figure 2 for the AUC and ROC plot. The average Matthews correlation was 0.36 (CI 0.1; 0.6).

Features were selected based on the LASSO feature selection penalty = 0.0. Features selected included height, age, X_C50_, Z_50_. Z*_f_*_c_, PhA_50_, and sex. The percentage drop in predictive performance accuracy when individual features were removed from the model was height 23% (CI 15.8; 31.7), age 13.7% (6.6; 21.4), X_C_ 5.3% (1.9; 9.3), Z_50_ 0.6% (CI 0; 2.8), PhA 0% (CI 0; 0), and sex 0% (CI 0; 2.3), as shown in Figure 2. This indicates that the model’s ability to identify children with oedema from new data would drop by 23% if height were excluded, as shown in Table 3. Repeated ninefold CV was performed with max repeats = 20. The software did not give a specific ratio of controls to cases in each fold, or the *K* value. Internal accuracy achieved by using only height and age was 70.6% (CI 61.6; 80.3); when adding the X_C_, the internal accuracy increased to 100% (CI 100; 100), as shown in Table 4. This means that the model could identify 7 out of 10 children with oedema using only height and age, but upon adding X_C_, the model was able to find 10 out 10 children in the available training set. The reported 100% internal accuracy refers to the accuracy achieved in specific cross-validation folds during model development. This metric reflects performance within training-derived partitions and does not represent the model's generalisation ability to unseen data. When applied to a hold-out test set, the final model achieved an area under the curve (AUC) of 0.84.

**Table 3:** The percentage decrease when excluding features from the prediction model.

Feature	Value added (%)	Lower confidence interval (%)	Upper confidence interval (%)
Ht	23.4	15.8	31.7
Age	13.7	6.6	21.4
X_C50_	5.3	1.9	9.3
Z_50_	0.6	0	2.8
Z*_f_*_c_	0	0	0
PhA_50_	0	0	0
Sex	0	0	2.3

Added value by including the feature in the final model with confidence intervals. X_C50_, reactance at 50 kHz; Z_50_, impedance at 50 kHz; Z*_f_*_c_, impedance at characteristic frequency; PhA_50_, phase angle as 50 kHz.

**Table 4:** Predictive performance when including one by one parameter in the model.

Feature	Predictive performance value (%)	Lower confidence interval (%)	Upper confidence interval (%)
Ht	70.6	61.6	80.3
Age	70.6	61.6	80.3
X_C50_	100	100	100
Z_50_	100	99	100
Z*_f_*_c_	100	99.4	100
PhA_50_	100	97.7	100
Sex	100	100	100

X_C50_, reactance at 50 kHz; Z_50_, impedance at 50 kHz; Z*_f_*_c_, impedance at characteristic frequency; PhA_50_, phase angle as 50 kHz.

For children with aNS, the model achieved a true positive rate of 0.92 (CI 0.88; 0.96). The model's precision, defined as correct positives out of the total positive population, was 0.84 (CI 0.80;0.89). Specificity, reflecting the proportion of correct negatives predicted, was 0.22 (CI 0.08;0.36).

### Model evaluation

The mean values of R were significantly lower for children with aNS compared to the HC group when using traditional statistical methods such as unpaired two-tailed *t*-tests, consistent with reference data (2). The model evaluated and included the specific features from BIA available measurements—X_C_, Z, and PhA—but not C_m_. The ML software tested C_m_, but it was not included in the final model.

## Discussion

In this study of eight children with aNS, automated ML software was able to construct a model with high accuracy for classifying healthy controls, but with low precision for classifying children with aNS. The model discriminated between the two classes, aNS and HC, with an AUC of 84%.

To the authors' knowledge, this is the first report to demonstrate the connection between alterations in disease status and raw impedance parameters in children with aNS, analysed using commercially available automated ML software for biological data. Importantly, the software only had BIA outcomes, which focus on the symptom of oedema, to assess whether it a child had aNS. Other researchers have employed ML models to predict disease status in children with aNS using clinical data. For example, Kou et al. (25) constructed a multivariate logistic regression model with the four clinical variables—erythrocyte sedimentation rate, suppressor T cells, D-dimer, and beta2-microglobulin—which correlated with steroid-resistant aNS in children with an AUC of 87%. Ye et al. (26) constructed a prediction model to evaluate the internal conditions and disease status of children with steroid-resistant aNS. They used eight clinical variables—including erythrocyte sedimentation rate, urine occult blood, percentage of neutrophils, immunoglobulin A, cholesterol, and vinculin autoantibody—and the model had an accuracy of 94%. Ye et al. and Kou et al. used clinical variables and not BIA outcomes and recruited a total of 91 and 111 subjects, respectively, with aNS and steroid-resistant aNS. Another study group used a dataset of 2,520 participants’ BIA data in an ML model to optimise intracellular fluid prediction with success in a healthy control group (27).

Research in healthcare ML and artificial intelligence is rapidly expanding, revealing potential applications across a range of medical fields (28, 29), such as interpreting chest radiographs (23), detecting cancer in mammograms (24), or detecting arrhythmias (30). Until now, very few well-validated ML models, usually developed from large datasets, have been deployed successfully in clinical practice (28, 31). The potential for faster data analysis and a more personalised treatment plans for patients with aNS could potentially be reached by employing ML, ultimately improving patient outcomes and optimising healthcare resources.

This pilot study assessed the feasibility of automated ML software in children suffering from severe oedema due to aNS. Ideally, ML software such as JADBio performs best with large datasets, up to millions, in variables. The small sample size was a limitation of this study, which poses the risk of over-fitting the model to the data. However, the low prevalence of paediatric NS, estimated to be around 2–5 per 100,000 (32), generally precludes the accumulation of large datasets. Thus, a large population would increase the model's ability to generalise, but this may not be achievable in real-life cohorts. While small cohorts may pose challenges due to limited data availability, it remains clinically important to prioritise research into this most common glomerular disease in childhood (33).

We excluded body weight from our model because it does not accurately estimate fluid volume or oedema in patients with aNS (5). This introduces a potential bias towards overestimating the ML model’s ability to diagnose children with aNS. The study included only one girl with aNS, which is not ideal for the software to model the effects of sex. However, the control group of 15 girls mitigates this limitation. The ML model incorporated sex as a parameter, but removing it would only reduce the model's predictive performance by 0−2.3%. Since BIA was conducted before puberty, when body composition differences are minimal, it is less likely that sex is an important factor to consider in diagnosing NS (34). While internal accuracy reached 100% when using height, age, and Xc_50_ alone in specific cross-validation configurations, the final model selected by the JADBio platform included additional features such as Z_50_, Z*_fc_*, PhA_50_, and sex. The model included PhA_50_, which showed a 0% drop in internal accuracy (CI 0; 2.3) when excluded from the model. We used a non-aggressive feature selection strategy, suitable for a pilot study. This meant that the model used all features that could contribute. This included sex and PhA_50,_ which may hold contextual or biological value. This was due to the application of a LASSO feature selection method, which allowed the inclusion of all features with marginal added value. This broader feature inclusion slightly reduced generalisation performance, as expected with small datasets.

Another potential limitation was including BIA data with only three pre-determined frequencies (5, 50, and 200 kHz) and *f*_c_. JADBio used the mean of two pre-determined frequencies measured by the BIA. With the predetermined frequencies, the software was able to employ a “working” model. However, a more precise model could be achieved by including the full range of impedance data from all measurement frequencies (50 within the range of 5 to 1,000 kHz) or complete frequency spectrum data obtained from Cole modelling. This would harness the potential of automated ML software to analyse large datasets and potentially enhance the true positive rate. Finally, the omission of other biomarkers, such as biochemical measurements or clinical symptoms, also limits the model and could be included in future studies.

Our findings highlight the need for further research using a larger dataset of children with NS, e.g., through multicentre trials and/or data sharing, and to include additional impedance data (at more frequencies) and clinical variables (e.g., blood chemistry). It may also be necessary to remove some features, as the LASSO penalty was set to zero. This resulted in the inclusion of all features with the possibility of adding value. However, both sex and PhA_50_ contributed no measurable improvement in predictive performance, meaning a 0% drop when excluded. As shown in Table 4, adding more features beyond a certain point can introduce noise into the model, potentially lowering its cross-validated performance accuracy. This reflects a known phenomenon in ML, where adding additional variables may disturb the model's internal structure, causing it to re-evaluate previous classifications and perform worse; generally, the most parsimonious model is preferred.

Finally, further work is needed to ascertain the importance of BIA measurements in ML models relative to clinical variables. ML models are excellent for predicting an outcome, but do not imply a causal inference (23). Additional studies are needed in larger datasets to develop a clinically useful prediction model and investigate the causal inference of using an ML model to predict NS and its complications in children.

## Conclusion

This cross-sectional study assessed the feasibility of automated ML software using BIA outcomes—PhA, IR, R, Z, X_C_, and C_m_—derived from impedance measurements obtained at specific frequencies. The ML software selected the BIA parameters X_C_, Z, and PhA, along with the clinical features height, age, and sex. A ridge logistic model achieved a high true positive rate (91.8%) but low specificity (21.7%) when classifying healthy controls and children with NSr. While specificity was too low to be acceptable in clinical settings, these findings suggest that the automated ML software can distinguish between the two groups, warranting further investigation in a larger cohort of children. JADBio shows promise as a tool for analysing complex biomedical data such as BIA, particularly in small datasets. The software focuses on the fully automated use of ML but does not provide fundamental information on why and how the final model is chosen. It functions as a “black box” in some situations, with limited insight into model construction and validation. Significantly, the model essentially discriminates oedema status rather than the aetiology of nephrotic syndrome, underscoring its potential applicability to other conditions characterised by altered fluid balance. The results, therefore, illustrate proof of concept rather than a diagnostic tool. This lack of transparency should be considered when interpreting results and evaluating their clinical relevance. If a larger study confirms the present pilot observations, the implementation of ML using BIA measures could potentially improve decision-making in selecting treatment pathways for children with NS. Future studies should include larger, independent cohorts, integrate biochemical and clinical markers, and explore the role of ML and BIA in body composition assessment and clinical decision-making.

## Data Availability

The raw data supporting the conclusions of this article will be made available by the authors, without undue reservation.
